# Dentate granule and mossy cells exhibit distinct spatiotemporal responses to local change in a one-dimensional landscape of visual-tactile cues

**DOI:** 10.1038/s41598-019-45983-6

**Published:** 2019-07-02

**Authors:** Dajung Jung, Soyoun Kim, Anvar Sariev, Farnaz Sharif, Daesoo Kim, Sebastien Royer

**Affiliations:** 1 Department of Biological Sciences, Korea Advanced Institute of Science and Technology, Daejeon, 34141 Republic of Korea; 2 Center for Functional Connectomics, Korea Institute of Science and Technology, Seoul, 02792 Republic of Korea; 3 Division of Bio-Medical Science and Technology, KIST School, Korea University of Science and Technology, Seoul, 02792 Republic of Korea

**Keywords:** Hippocampus, Spatial memory

## Abstract

The dentate gyrus (DG) is critical for detecting changes in environments; however, how granule cells (GCs) and mossy cells (MCs), the two excitatory cell types of the DG, respond to small changes in the object layout is unclear. Here, we recorded GCs and MCs, identified by spike feature and optogenetic tagging, as mice ran on a treadmill belt enriched with visual-tactile cues. We observed that fixing a new cue on the belt induced a reconfiguration of GC and MC spatial representations via the emergence, extinction and rate alteration of firing fields. For both GCs and MCs, the response was maximal near the cue and spread over the entire belt. However, compared to the GC response, the MC response was stronger and more immediate, peaked at a slightly earlier belt position, and exhibited a transient component reminiscent of neuromodulatory activity. A competitive neural network model reproduced the GC response contingent on both the introduction of new object-vector inputs and the reconfiguration of MC activity, the former being critical for spreading the GC response in locations distant from the cue. These findings suggest that GCs operate as a competitive network and that MCs precede GCs in detecting changes and help expand the range of GC pattern separation.

## Introduction

The dentate gyrus (DG) is largely hypothesized to implement a ‘pattern separation’ operation^1–4^ allowing similar but not identical input patterns from the entorhinal cortex (EC) to be distinctly encoded in CA3. Specifically, the DG network is believed to generate distinct activity patterns for slightly distinct EC inputs, and the supply of this differentiated information to CA3 is believed critical to prevent the merging of information by CA3 pattern completion^2,4–6^. While lesion studies largely support this hypothesis^7–10^, how pattern separation is implemented by the DG remains unclear.

One behavior paradigm that requires intact DG function is the detection of small changes in the spatial layout of objects in an environment. Animals tend to spend more time exploring the location of displaced objects; however, they do not show such behavior when DG functions are impaired^8,10^. How the activity of DG cells is altered by small changes in object layout is largely unknown. Yet, we can envision that several factors might compel the DG to produce distinct representations. First, granule cells (GCs) are believed to be especially predisposed to produce sparse and orthogonal cell assemblies^2,11,12^ due to a strong inhibition that promotes competition between GCs^13^ and to extensive divergence of EC-to-GC afferents, as GCs outnumber EC cells by a ratio of at least 5:1 (possibly reaching 10:1)^14,15^. Second, GC place fields are suggested to be contingent on both object and spatial information^9,16,17^ and therefore sensitive to object displacements. Activity patterns related to objects, boundaries and path integration are reported in the lateral (LEC) and medial (MEC) divisions of the EC^18–22^. Specifically, GC place fields may emerge from the conjunction of MEC grid cells^18,22,23^, MEC object-vector cells^24^ and LEC object-specific cells^21^. Third, mossy cells (MCs) exhibit activity patterns sensitive to changes in local cues^25,26^ and are hypothesized to convey a novelty signal, which could help transform GC representations via MC-to-GC excitation and disynaptic inhibition^27–29^.

Despite the importance of an intact DG for detecting changes in the spatial layout of objects^7–10^, experiments that examine DG cell responses to small changes in object layout are missing. Here, we used silicon probes with integrated light guides^30,31^, two mouse lines (POMC-Cre and DRD2-Cre) expressing Chronos^32^ in GCs and MCs^25,27,33,34^, and a treadmill apparatus on which mice ran head-fixed through a sequence of visual-tactile cues fixed on the belt^35–37^. We identified putative GCs and MCs and examined their spatial and temporal responses to the addition of a visual-tactile cue to the familiar layout of the belt. We implemented a computational model of the GC network that incorporated grid cell, object-vector cell, MC and feedback inhibitory inputs, and evolved via Hebbian synaptic modifications^13,38,39^. We showed that spatial representations reconfigure differently for GCs and MCs in ways that are consistent with MCs detecting changes first, GCs operating as a competitive network, and MCs enabling the spreadout of GC remapping in locations distant from the cue.

## Results

### Recording and photostimulation of dentate units on the treadmill

**Figure 1:**
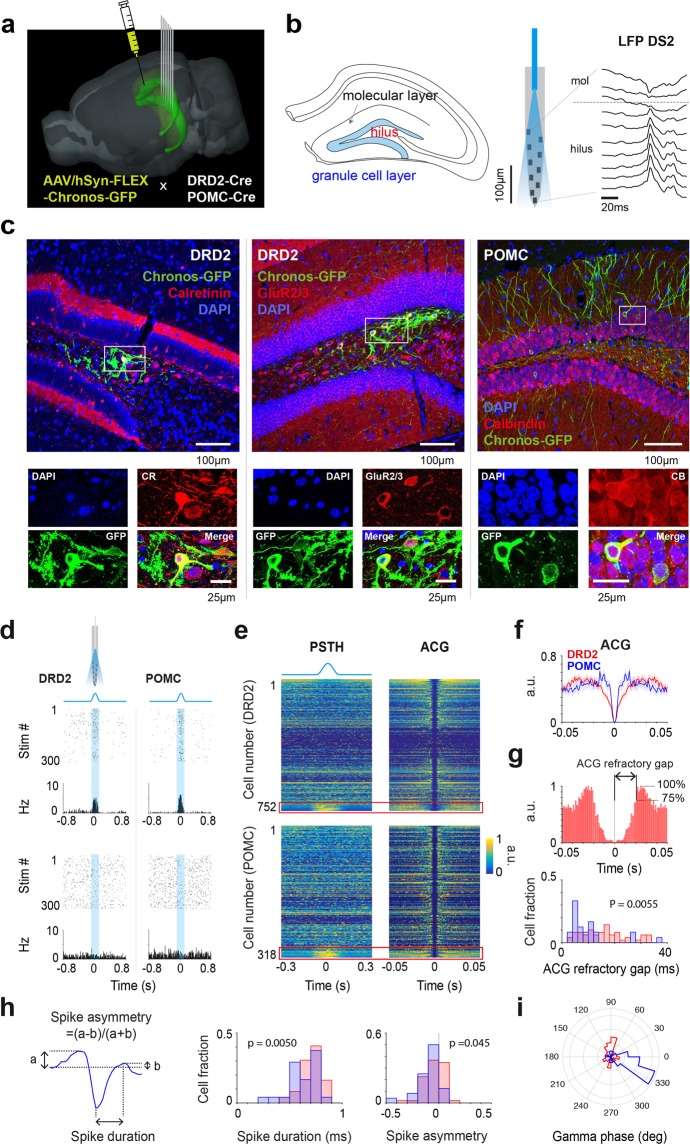
Optogenetic excitation of GCs and MCs. (**a**) 3D representation of the mouse brain (Allen Mouse Brain Institute; www.alleninstitute.org) showing recording electrode configuration in the dorsal dentate gyrus of DRD2-Cre and POMC-Cre mice injected with AAV/hSyn-FLEX-Chronos-GFP. (**b**) Left, scheme showing the location of the hilus and granule cell layer on a coronal section of the hippocampus. Right, layout of recording sites and optic fiber for a shank of the silicon probe and profile of a local field potential dentate spike 2 (LFP DS2). (**c**) Low-magnification images, confocal images of GFP-virus expression (green) and DAPI staining (blue), together with immune-staining (red) for calretinin (left) and GluR2/3 (middle) in DRD2-Cre and for calbindin (right) in POMC-Cre mice. The rectangles indicate the regions selected for the high-magnification images shown below. High-magnification images, DAPI, GFP and calretinin (or GluR2/3 or calbindin) signals shown separately and merged. (**d**) Examples of the cell response (raster and peri-stimulus histogram (PSTH)) to light stimuli (473 nm wavelength, ~50 µW amplitude, 100-ms-duration single sine waves), for a light-excited cell (upper) and an unaffected cell (lower) in DRD2-Cre (left) and POMC-Cre (right) mice. (**e**) Color-coded representations of cell PSTH (left) and spike autocorrelograms (ACGs) (right) for DRD2-Cre (upper) and POMC-Cre (lower) mice. Cells are represented in the rows of the matrices and ordered according to the magnitude of light responses. Red box indicates the light-excited cells, i.e., cells with light response Z-score > 5 (DRD2, n = 49 cells; POMC, n = 25 cells). (**f**) Average cell ACG for DRD2 (red) and POMC (blue) light-excited cells (line, average; shadow, s.e.m). Note the wider profile of the average ACG for DRD2 light-excited cells. (**g**) Upper, ACG of a DRD2 light-excited cell and illustration of the measure ACG refractory gap (defined as the duration for the ACG to reach 75% of peak value). Lower, distribution of ACG refractory gap values for DRD2 (red) and POMC (blue) light-excited cells (mean ± s.e.m; DRD2, 15.5 ± 1.2 ms; POMC, 9.8 ± 1.7 ms; p = 0.0055, unpaired t-test). (**h**) Example of spike waveform and illustration of how spike duration and asymmetry are measured (left) and distribution of spike duration (middle, DRD2, 0.7 ± 0.01 ms; POMC, 0.6 ± 0.03 ms; p = 0.0050, unpaired t-test) and spike asymmetry (right, DRD2, −0.05 ± 0.01; POMC, −0.1 ± 0.02; p = 0.045, unpaired t-test) values for DRD2 (red) and POMC (blue) light-excited cells. (**i**) Distribution of cell-preferred gamma phases for DRD2 (red) and POMC (blue) light-excited cells.

AAV/hSyn-Flex-Chronos-GFP was injected into the DG of 8 mice (5 DRD2-Cre and 3 POMC-Cre mice) to selectively express Chronos in MCs (n = 5) or in GCs (n = 3). Following a period of 7 days to recover from surgery, the mice were trained for 3 weeks to run head-fixed on a 211-centimeter-long treadmill belt enriched with visual-tactile cues, with water rewards provided via a lick port on every trial (belt cycle) at a fixed position of the belt. Then, for simultaneous recordings of electrophysiological activity and photoexcitation of DG neurons, a silicon probe with integrated optical fibers^30,31^ was chronically implanted in the dorsal DG (Fig. 1a). The electrode position in the DG was assessed by the profile of local field potential (LFP) type 2 dentate spikes (DS2s), whose polarity is positive in the hilus and reverses above the granule cell layer^25,40^ (Fig. 1b), and confirmed by the concentration of recorded cells below the DS2 reversal point and the detection of optogenetic cell responses. For DRD2-Cre mice, GFP-labeled cells were visible in the hilus and colocalized with the MC markers calretinin and GluR2/3^26,27,33,41–43^, while for POMC-Cre mice, GFP-labeled cells populated the GC layer and colocalized with the GC marker calbindin^27,44,45^ (Fig. 1c). POMC is typically used as a marker for immature GCs because its expression is limited to the 1-month period following cell mitosis^46^. While we cannot be sure, Chronos expression may have been biased toward mature adult-born GCs (>5–6-week old^47^) at the time of the recordings 4 weeks after virus injection.

To excite GCs and MCs that expressed Chronos, a series of sine-wave-shape photostimuli (>300 single sine waves, 50 µW, 100-ms duration, every 3 s) was delivered. Compared to a short pulse, such a gradual stimulus waveform is more likely to induce asynchronous spiking as cells reach spike thresholds at distinct phases of the stimulus (because of differences in excitability, proximity to the light source and virus expression), thereby improving spike detection^30,48,49^ and reducing the chance of indirect cell excitation. The peri-stimulus histograms of a fraction of cells showed excitatory responses (Fig. 1d,e). For the next analysis, we considered excitatory responses having a Z-score > 5 (comparing the peak of the response with the 1-s period preceding the stimulus; DRD2: 49 out of 752 cells; POMC: 25 out of 318 cells).

### Identification and characterization of putative GCs and MCs

To allow the identification of putative GCs and MCs based on physiological criteria, we searched differences in spike features for DRD2 and POMC light-excited cells (Fig. 1f–i). First, we found that spike autocorrelogram profiles were characteristic of short-interval burst activity for POMC light-excited cells and showed large humps flanking a wide refractory gap for DRD2 light-excited cells (Fig. 1f), consistent with the GC and MC autocorrelogram profiles obtained from *in vivo* intracellular recordings^50^. To quantify this difference, we measured an ACG refractory gap, defined as the duration for the autocorrelogram to reach 75% of its peak value, for each cell (Fig. 1g). As expected, DRD2 light-excited cells had, on average, higher ACG refractory gap values than POMC light-excited cells (Fig. 1g; DRD2, 15.5 ± 1.2 ms; POMC, 9.8 ± 1.7 ms; p = 0.0055, unpaired t-test). Furthermore, compared to DRD2 light-excited cells, POMC light-excited cells had shorter spike durations (Fig. 1h; DRD2, 0.7 ± 0.01 ms; POMC, 0.6 ± 0.03 ms; p = 0.0050, unpaired t-test) and more negative spike asymmetry values (Fig. 1h; DRD2, −0.05 ± 0.01; POMC, −0.1 ± 0.02; p = 0.045, unpaired t-test). Finally, POMC light-excited cells showed a preference to discharge before the troughs of local field potential gamma oscillations (30–80 Hz; measured in the hilus), while DRD2 light-excited cells showed no clear bias (Fig. 1i).

**Figure 2:**
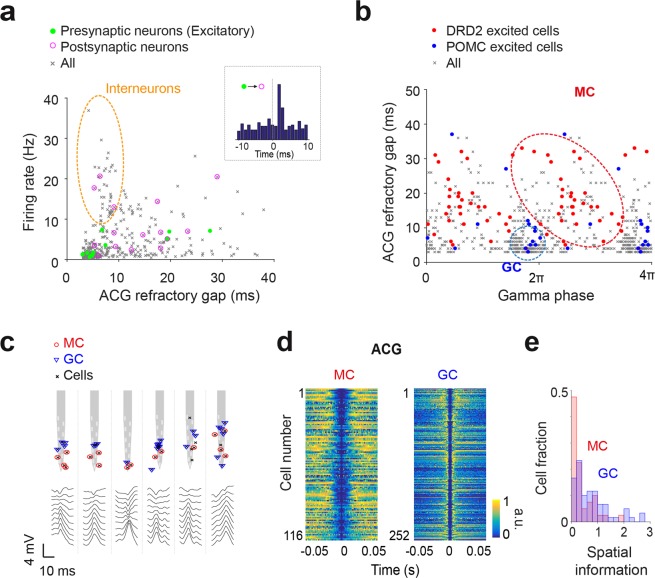
Identification of putative MCs and GCs. (**a**) Distribution of cells according to firing rate and ACG refractory gap. Green dots, excitatory cells identified by a large peak at monosynaptic latency (<3 ms) in short-time cross-correlograms of a neuron pair^51^ (inset). Magenta circles, neurons receiving excitation from identified excitatory cells. Orange ellipsoid, putative inhibitory interneurons segregated by high firing rate, short ACG refractory gap and lack of identified excitatory neurons. (**b**) Clustering of neurons by cell-preferred gamma phases and ACG refractory gap. Putative inhibitory cells identified in (a) are excluded. Red dots, light-excited cells in DRD2-Cre mice. Blue dots, light-excited cells in POMC-Cre mice. Red and blue ellipsoids, putative MCs (n = 116 cells) and GCs (n = 252 cells), respectively. (**c**) Examples of shanks on which both MCs and GCs were recorded, showing (upper) recording sites, positions of MCs (red circles) and GCs (blue triangles), and (lower) LFP DS2. Notice that MC positions match the positivity of the LFP DS2 (in the hilus) and that GCs tend to be located above, closer to the reversal of the LFP DS2. (**d**) Color-coded representation of autocorrelograms for MCs (left) and GCs (right). (**e**) Spatial information for MCs (red) and GCs (blue) (p = 7.03e-04, Wilcoxon rank-sum test).

The light stimuli allowed only the detection of a subset of GCs or MCs in a mouse. To identify all putative GCs and MCs in all mice, we measured the above spike features for all cells and examined the overlaps with the spike features of POMC/DRD2 light-excited cells^25^ and putative excitatory neurons (identified from cell-pairs cross-correlogram analysis^51^). We first excluded a group of cells categorized as putative interneurons based on their high firing rates, low ACG refractory gap values, and the lack of overlap with putative excitatory neurons (Fig. 2a). Then, we found that the combination of features that best separated POMC and DRD2 light-excited cells was the cells’ ACG refractory gap together with the cells’ preferred gamma phase. Putative GCs (n = 252) were characterized by a narrow ACG refractory gap, a preference to discharge during the troughs of gamma oscillations and an overlap with POMC light-excited cells (Fig. 2b,d, Right). In contrast, putative MCs (n = 116) were characterized by a wide ACG refractory gap, a preference to discharge at other phases of gamma oscillations and an overlap with DRD2 light-excited cells (Fig. 2b,d, Left).

Next, we examined the relative position of putative GCs, MCs and local field potential (LFP) type 2 dentate spike (DS2)^25,40^ along the electrode shanks (Figs 1a and 2c; See methods). Consistent with anatomical data, putative GCs were located closer to the site of DS2 polarity reversal, which is located above the granule cell layer, while putative MCs were shifted toward the positivity of the DS2, i.e., toward the hilus (Fig. 2c). As previously reported^25,43^, putative GCs had higher spatial information compared to putative MCs (Fig. 2e; p = 7.03e-04, Wilcoxon rank-sum test).

### Addition of a landmark

The next experiment was performed with 5 of the 8 mice (2 DRD2 and 3 POMC). For the analyses, we considered cells with a mean firing rate > 0.5 Hz and stable place fields (for which spatial correlation between session halves > 0.5; GC, n = 40; MC, n = 40), and POMC and DRD2 data were pooled and analyzed together, as we observed no qualitative difference between the two groups.

**Figure 3:**
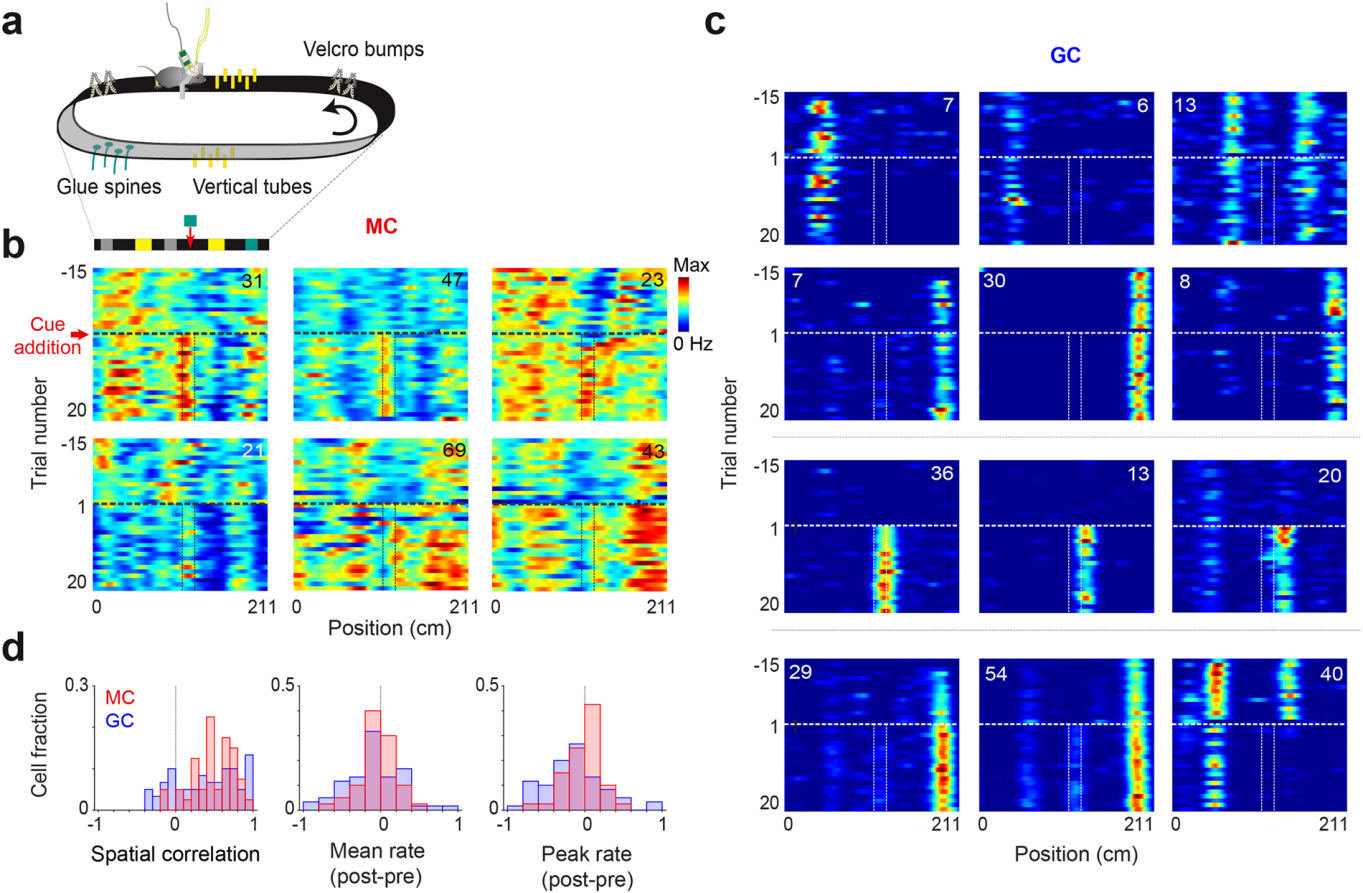
Alteration of cell firing fields by the introduction of a cue to the treadmill belt. (**a**) Schematic representation of the treadmill setup and layout of visual-tactile cues on the belt. (**b**) Single trial rate maps for six MC examples, showing 15 trials before and 20 trials after the cue addition. Dash line, time of the cue addition. Plain lines, edges of the added cue. (**c**) Same as (**b**) for GCs showing (2 upper rows) little changes, (3rd row) emergence of a place field, and (4th row) change in firing rate. (**d**) MC and GC distribution for spatial correlation, change in mean firing rate (MC versus GC, p = 0.0037, F-test) and change in peak firing rate (MC versus GC, p = 2.91e-04, F-test) between trials pre and post cue addition.

In a second recording session in which no photostimuli were delivered, we fixed a new visual-tactile cue on the belt after 10 to 15 trials. We first examined individual cell responses by plotting firing rate maps for trials before (pre) and after (post) the object addition (Fig. 3b,c) and by computing spatial correlations (pixel-by-pixel correlation coefficient) and firing rate changes between ‘pre’ and ‘post’ rate maps (Fig. 3d). GCs showed a range of responses. While some GCs were not much affected, other GCs developed a place field near the object position or exhibited changes in firing rate (Fig. 3c). Most MCs were affected by the manipulation (Fig. 3b), as only a small fraction of MCs showed very high spatial correlation (Fig. 3d Left). However, MC alterations were more partial than GC alterations, as MCs showed fewer spatial correlations that were very low and exhibited, on average, smaller changes in firing rate (Fig. 3d; mean rate, p = 0.0037; peak rate, p = 2.91e-04, F-test).

### Spatiotemporal profiles of changes for GC and MC firing rates

**Figure 4:**
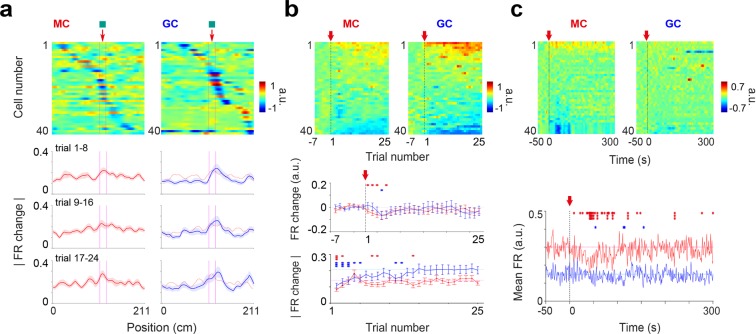
Spatiotemporal profiles of changes for GC and MC firing rates. (**a**) Color-coded panel, firing rate change by position for individual cells, using pre trials as references. Cells are sorted by the position of maximum change (in absolute value). Lines, average firing rate changes (absolute values) by position for blocks of 8 trials, with MC averages superimposed to GC averages (right panels) to facilitate comparison (MC, n = 40 cells, GC, n = 40 cells). Note that most changes are near the added cue. (**b**) Change in normalized firing rate over trials, using pre trials as reference. Color-coded panel, individual cells sorted according to firing rate changes. Middle, population average (lines) and s.e.m (error bars) for firing rate change (significance relative to 0). Note that population firing rates are on average preserved. Bottom, absolute values of changes (significance relative to trials 21–25). Note the progressive development for GCs. (**c**) Change in firing rate (normalized) over time, using the 50-s time period preceding cue addition as reference. Color-coded panel, individual cells sorted according to firing rate changes. Bottom, population mean firing rates (significance relative to pre trials). *p < 0.05, **p < 0.01, ***p < 0.001, unpaired t-test.

To examine the spatiotemporal profiles of GC and MC responses, we first examined how firing rates changed depending on belt positions, trials and time. To examine the spatial profile of firing rate changes, we computed firing rate changes for each position of the belt using ‘pre’ rate maps as references and implemented color-coded matrices displaying individual cells as well as cell population averages (Fig. 4a). For both individual cell and population average displays, firing rate changes were seen throughout the whole belt; however, the largest effect was in the vicinity of the added cue (average change within cue versus > 10 cm away from cue; MC: 0.21 ± 0.02 versus 0.15 ± 0.007, p = 0.010; GC: 0.21 ± 0.03 versus 0.13 ± 0.01, p = 0.008, paired t-test). Furthermore, the peak of the response was shifted to after the cue for GCs, whereas the peak was aligned with the cue for MCs (MC: −0.4 ± 1.8 cm from middle of cue; GC: +6.6 ± 2.2 cm from middle of cue; p = 0.021, unpaired t-test; cell averages using cells with peak changes <10 cm away from the cue).

To examine the dynamics of cell responses, we computed trial-by-trial changes in firing rate for individual cells using ‘pre’ rate maps as references and implemented color-coded matrices displaying individual cells as well as cell population averages (Fig. 4b). In both GC and MC populations, firing rates were increased for a subset of cells while decreased for another (Fig. 4b, color-coded). However, at the population level, the average cell firing rates were relatively unaltered (Fig. 4b, middle), suggesting that the firing rate elevations and reductions were relatively balanced in both the GC and MC populations. Therefore, we also computed average firing rate changes using the absolute values of changes for each cell (Fig. 4b, bottom). GCs and MCs showed distinct dynamics. While the magnitude of GC firing rate changes progressively increased and reached a plateau value after 15 trials, the MC response was more immediate, reaching a plateau value after 4 trials.

Interestingly, an initial transient decrease was visible in the population average firing rates (Fig. 4b, middle). To precisely examine this transient component, we computed cell firing rate changes over a 300-s time period using the 50-s period preceding the cue addition as a reference and implemented color-coded matrices displaying individual cells as well as cell population averages (Fig. 4c). A transient decrease in firing rate, lasting approximately 1 minute, was visible for some MCs (Fig. 4c, color coded) and detectable in the MC population average (Fig. 4c, bottom). Such an effect is reminiscent of the one-minute transient inhibition of hilar cells observed following the stimulation of the locus ceruleus^52^.

### Spatiotemporal profiles of changes for GC and MC population vectors

**Figure 5:**
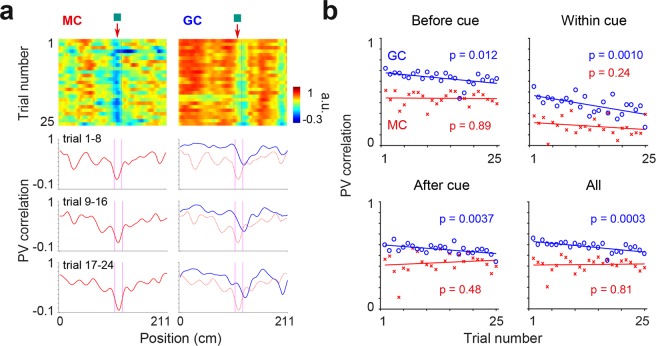
Spatiotemporal profiles of changes for GC and MC population vectors. (**a**) Color-coded panels, population vector (PV) correlation calculated in each belt position and for each trial after cue addition, using pre trials as references (MC, n = 40 cells, GC, n = 40 cells). Lines, PV correlation calculated in each belt position for blocks of 8 trials, using pre trials as references. The PV correlations of MCs are superimposed to the PV correlations of GCs (right panels) to facilitate comparison. Note the peak alteration near the added cue and the shift between GC and MC peaks. (**b**) Average PV correlation for positions before cue (from 0 to 109 cm), within cue (from 109 to 124 cm), after cue (from 124 to 211 cm) and all positions, as a function of trials (p-values, Pearson correlation).

We next examined how population vectors^25,53^ were altered depending on belt position and trials. For this, we computed MC and GC population vector correlations for each position of the belt and for either single trials or block of 8 trials, using ‘pre’ rate maps as references (Fig. 5a). While population vectors were altered throughout the whole belt for both MCs and GCs, the alteration peaked in the vicinity of the cue (within cue versus > 10 cm away from cue; MC: 0.2 ± 0.04 versus 0.6 ± 0.01, p = 0.78e-23; GC: 0.6 ± 0.05 versus 0.8 ± 0.01, p = 1.57e-09; paired t-test) and the peak was shifted to after the cue for GCs but aligned with the cue for MCs (Fig. 5a; MC: 0.4 ± 1.2 cm from middle of cue; GC: +9.3 ± 0.7 cm from middle of cue; p = 5.50e-07, paired t-test).

Importantly, the alteration of the population vector was stronger for MCs than for GCs, a phenomenon that was observed in all positions of the belt initially (Fig. 5a; trial 1–8 population vector correlation before cue: GC, 0.7 ± 0.01, MC, 0.5 ± 0.03, p = 3.5e-05; within cue: GC, 0.4 ± 0.02, MC, 0.2 ± 0.03, p = 2.5e-04; after cue: GC, 0.6 ± 0.01, MC, 0.4 ± 0.05, p = 0.01; unpaired t-test). However, the difference was reduced over trials as GC population vector correlation progressively decreased while MC population vector correlation was unchanged, a phenomenon that was also observed in all positions of the belt (Fig. 5b; Before cue: GC, p = 0.012, MC, p = 0.89; Within cue: GC, p = 0.0010, MC, p = 0.24; After cue: GC, p = 0.0037, MC, p = 0.48; All positions: GC, p = 0.0003, MC, p = 0.81; Pearson correlation coefficient).

### Modeling of the GC response to cue addition

**Figure 6:**
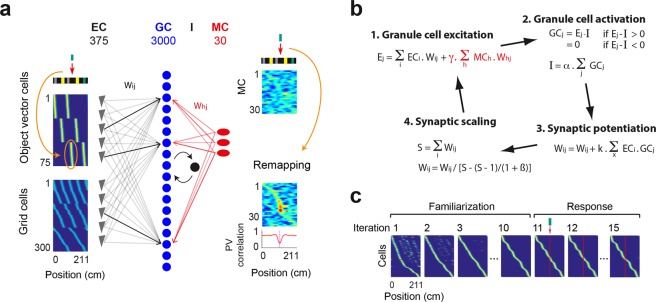
Competitive learning model of the DG. (**a**) Model of the DG network. GCs receive excitatory inputs from EC grid cells, EC object-vector cells and MCs, and are subject to feedback inhibition. The EC-to-GC and MC-to-GC synaptic weight matrices are referred to as Wij and Whj respectively. The threshold used as feedback inhibition is referred to as I. Color-coded panel, rate maps of object-vector cells (upper left), grid cells (lower left), and MCs (right). Object-vector cells encode various distances to each object, with a bias toward positions after the cues, while grid cells have various phase and periodicity. MCs have several fields randomly distributed on the belt. The orange arrows indicate the changes in EC and MC activity patterns upon the object addition. For EC cells, a new set of object-vector firing fields is added (orange ellipsoid). For MCs, the amplitude for various firing fields is randomly altered, but the degree of alteration is larger near the added cue such that the MC population vector shows a similar alteration profile as in the data. (**b**) All the steps executed during one model iteration. (1) The excitation Ej received by the GCj in a given position. (2) The levels of GCj activation and feedback inhibition I in that position. The value of I is estimated numerically, by finding among a range of I values the one that best satisfies the two relations. (3) The potentiation of synaptic weights, proportional to the level of EC-GC pair co-firing throughout the belt. (4) The scaling of synaptic weights, with a rate determined by the parameter ß. (**c**) GC rate maps over model iterations. Only the active GCs are represented and ordered according to firing field position. The first 10 iterations were executed with faster learning parameters (k = 20 and ß = ∞) to reflect the several day period of familiarization to the belt. Then, learning parameters were reduced (k = 1 and ß = 3) to produce progressive changes over trials.

The dentate gyrus is often modeled as a competitive network in which discrete place field representations are produced through ‘competitive learning’, that is, through the combination of Hebbian synaptic plasticity and GC competition^13,38,39,54^. Therefore, we tested whether GC responses to cue addition could be replicated by a competitive network model of the DG in which place field representations were initially developed through competitive learning. We incorporated only the mechanisms necessary to replicate the response features of experimental data in the model. The model was therefore simplistic. Briefly, 3000 GCs received excitatory inputs, with randomly assigned synaptic weights, from 300 EC grid cells, 75 EC object-vector cells and 30 MCs, and were subject to feedback inhibition (Fig. 6a, See methods). The firing patterns of the inputs were generated with Gaussian functions and were periodic for grid cells, matched the positions of a pair of cues (slightly shifted toward after the cues) for object-vector cells and were randomly distributed for MCs. The activity of a GC in a given position was the weighted sum of EC and MC activity minus the inhibition. The inhibition was proportional to the summed activity of GCs. In each model iteration, the EC-to-GC synapses were modified via synaptic potentiation and scaling mechanisms (Fig. 6b).

**Figure 7:**
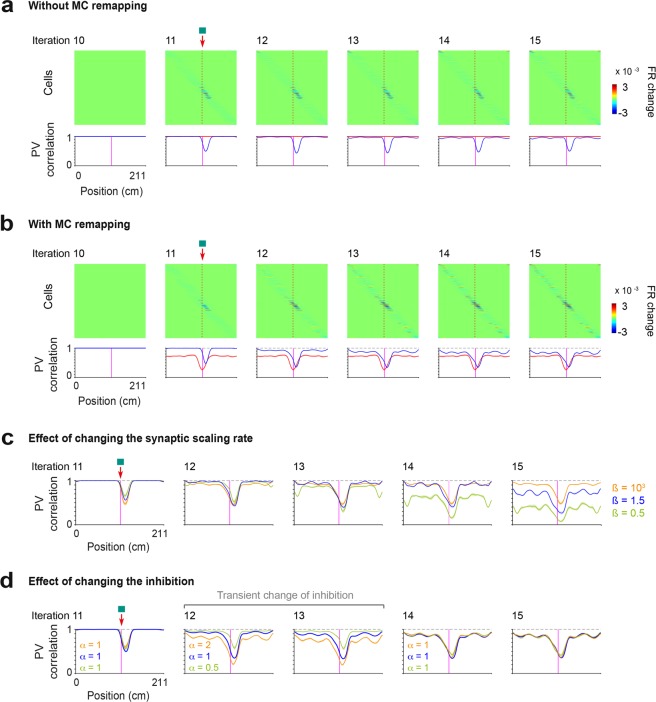
Modeling of GC response. (**a**) The effect of the addition of the new set of object-vector firing fields in the model, without MC remapping. Color-coded panel, change in firing rate for individual GCs over iterations following the cue addition, using iteration 10 as the reference. Cells are sorted by the position of maximum change (in absolute value). Lines, population vector correlation for MCs (red) and GCs (blue). Note that the alteration of the GC population vector near the cue is reproduced, but that alteration in positions distant from the cue is lacking, which is not consistent with the experimental data. (**b**) Same as (**a**) except that MC remapping was implemented together with the addition of the new set of object-vector firing fields. Note that the GC population vector is altered in all positions and that the alteration near the cue has a skewed profile, consistent with the experimental data. (**c**) GC population vector correlation over iterations for 3 different synaptic scaling rates, ß = 10^3^ (orange), ß = 1.5 (blue) and ß = 0.5 (green). Note that for ß = 10^3^ (i.e., the sum of synaptic weights is almost normalized), no progressive development in population vector correlation is observed over time, while for ß = 0.5 (i.e., small scaling of synaptic weights per iteration), the population vector correlation strongly decreases over iterations. (**d**) GC population vector correlation over iterations illustrating the effect of transiently changing the inhibition parameter α, during the iterations 12 and 13. Notice the decrease and increase in population vector correlation when the parameter α is decreased to 0.5 (green) and increased to 2 (orange), respectively.

Following 10 iterations that produced a continuous spread of single GC place fields^39^ (Fig. 6c), the cue was added. To simulate the addition of the cue, a new firing field was added at the position of the cue for the set of object-vector cells already encoding the other position of the cue (Fig. 6a). We first examined the effect of this simple manipulation on GC firing rates and population vector, using iteration 10 as a reference. Similar to the experimental results, the GC population vector and firing rates were mostly affected in the vicinity of the added cue, and both negative and positive changes in firing rate were observed (Fig. 7a), suggesting that these features could be explained simply by the addition of object-vector cell inputs and the competitive interaction between GCs. However, in contrast to experimental results, the GC population vector was almost not affected in locations distant from the cue. We therefore asked if altering MC activity might help reproduce this feature. To test this, in addition to the addition of object-vector cell inputs, we altered MC firing activity throughout the entire belt in an incremental manner toward the cue such that the profile of MC population vector correlation resembled the one of experimental data (Figs 6a and 7b, see Methods). With this additional mechanism, the model could also reproduce the GC population vector correlation in locations distant from the cue (Fig. 7b). Moreover, the GC population vector correlation developed a skewed profile in the vicinity of the cue, similar to that observed in the experimental data.

Importantly, a critical factor to reproduce the progressive aspect of GC population vector changes was a partial scaling of synaptic weights per iteration. For the value of the parameter ß = 3 used so far (Fig. 6b), the sum of synaptic weights was shifted closer (but not made equal) to 1 on each iteration, and the GC population vector correlation showed a gradual development similar to that observed in the experimental data. However, such gradual development could not be reproduced when the values of ß were much higher or lower, i.e., when synaptic scaling operation leaned toward normalization or no scaling, respectively (Fig. 7c). A partial scaling of synaptic weights per iteration is consistent with the gradual restoration of total synaptic weight observed after synaptic potentiation *in vitro*^55^.

Finally, because MCs displayed transient changes in firing rate and are suggested to contribute feedforward inhibition to GCs^52,56,57^, we examined the effect of transiently modulating the inhibition parameter α. Interestingly, increasing and reducing α changed the GC population vector correlation in a similar direction (Fig. 7d). Hence, the degree of GC remapping can be dynamically tuned via the modulation of inhibition, and MCs are well positioned to control such operation.

## Discussion

Despite the critical involvement of the DG in detecting changes in object locations^8,10,19^, the impact of local changes in object layout on DG cell activity has not been investigated. With the combination of silicon probe recording, optogenetic techniques and running behavior on a cue-enriched treadmill, we identified putative GCs and MCs and monitored their trial-by-trial responses to the introduction of a local cue.

The method used to identify putative GCs and MCs differed from those used in previous studies^25,42,43^ and presented both potential advantages and limitations. We used Cre-dependent virus injections in DRD2-Cre and POMC-Cre mice to express Chronos in MCs and GCs, respectively. Virus expression in DRD2-Cre mice was reported to be quite specific to MCs in the DG but present in interneurons of the CA hippocampal regions^58^. Additionally, Chronos expression may have been biased toward mature adult-born GCs^47^ because POMC expression is limited to the 1-month period following cell mitosis^46^ and because recordings were carried out 4 weeks after virus injection such that a large fraction of virus-infected GCs were likely mature (>5–6 week old^47^) by the time of the recordings.

We used sine-wave-shape light stimuli to excite GCs and MCs expressing Chronos. This type of stimulus was used for several reasons. First, long sine waves have been shown to be better than short pulses for the detection and isolation of light-triggered spikes^30^. Indeed, short light pulses tend to produce synchronous cell activation, generating an overlap of spike waveforms of cells that compromise spike sorting. In contrast, with the progressive change in light intensity generated by the sine wave, cells are likely to reach spike thresholds at distinct phases of the stimulus because of differences in excitability, proximity to the light source and Chronos expression. Second, the asynchronous cell activation generated by the sine wave should reduce the chance of disynaptic cell activation. Third, light excitation of cells is in some respects better than light silencing of cells (using halorhodopsin^59^ for instance). To detect the effect of cell silencing, the baseline firing rates of cells should be high, which is not the case for GCs^11,60,61^. Additionally, while the possibility of an indirect effect exists in both approaches, the effect of light excitation is more easily observed in real time than that of light silencing, allowing for the adjustment of the light intensity to minimal levels (to reduce indirect effects).

We found that the two features providing the best separation for putative GCs and MCs, based on POMC and DRD2 light-excited cell data, were the spike autocorrelogram profile and spike gamma phase of cells. The wide profile of MC spike autocorrelograms was consistent with *in vivo* intracellular recording data in which MCs were identified via intracellular labeling^50^. Additionally, a difference in spike gamma phase between GCs and MCs was observed in another study^25^, but to a smaller extent. This discrepancy might not necessarily reflect a difference in cell classification but differences in the locations where local field potential gamma oscillations were measured. Finally, the differences in MC and GC activity patterns and spatial responses were largely consistent with other studies reporting that MCs have wider and more numerous firing fields and show stronger remapping than GCs^25,42,43^.

With the addition of a local cue on the belt of the treadmill apparatus, we could observe both temporal and spatial aspects of cell responses. The GC response was characterized by a reconfiguration of place fields that peaked in the vicinity of the added cue. This local peak likely arose from the addition of local object-vector inputs, as illustrated by the model. Interestingly, the peak was shifted slightly after the cue, possibly because of a similar shift of object-vector representations or local network computations. Despite the additional cue and the reconfiguration of spatial representations, the overall firing activity of the GC population was relatively unchanged as the increase and decrease in cell activity were relatively balanced, suggesting the existence of mechanisms preserving the scale of GC activity. Furthermore, changes in GC population activity developed progressively over trials (Figs 4 and 5), suggesting the involvement of synaptic plasticity mechanisms. The model reproduced this feature using two types of synaptic plasticity mechanisms: synaptic potentiation akin to Hebbian long-term potentiation (LTP)^54,62^ and synaptic scaling, which is necessary to prevent exponential growth of synaptic weights^13,38,39^. While LTP appears immediately after LTP inducing stimuli^54,55,63^, synaptic scaling is a slower process, developing over 30 minutes *in vitro* via heterosynaptic depression^55^ and partly taking place during slow-wave sleep/sharp-wave ripple activity *in vivo*^64,65^. Consistent with this time scale, the rate of synaptic scaling had to be relatively low for the model to reproduce the progressive decrease in GC population vector correlation.

MCs are suggested to play a role of sentinels that inform GCs of object layout changes by deciphering changes from semilunar GC inputs^66–69^. The differences between MC and GC responses were consistent with this hypothesis. First, the MC response was stronger^25,43^, more immediate, and peaked earlier on the belt than the GC response (Figs 4 and 5), consistent with MCs not being controlled by GC-to-MC inputs and MCs preceding GCs in detecting change. Second, in contrast to GCs, several MCs displayed a one-minute-long transient response (Fig. 4c). One-minute-long cell responses have been reported in the hilus for stimulations of the locus ceruleus^52^, a structure that shows phasic activation to change in environmental contingencies^70,71^ and releases both noradrenaline^72^ and dopamine^73^. Although hilar cells were generally called interneurons in this study, MCs, the most common cell type in the hilus^74^, likely expressed such responses, considering that the large majority of recorded cells did. The transient response we observed for MCs might therefore be generated by a novelty-related neuromodulatory signal from the locus ceruleus. Hence, consistent with the sentinel hypothesis^27^, MCs were not controlled by GC-to-MC inputs and likely received novel cue information from other afferents, such as semilunar GCs^27,66–68,75^, direct EC afferents^28,76^ and the locus ceruleus. MCs could then contribute to changes in GC encoding via both direct and indirect inputs to GCs^5,27,28,74,75^.

An alteration of both MC and GC population vectors was visible in locations distant from the added cue, a feature that did not arise from noise as it developed over time (Fig. 5). While the mechanism underlying this feature is unclear for MCs, a possible scenario is that it originates from the abovementioned transient neuromodulatory effect, which should overlap with all belt positions due to its minutes-long duration. Importantly, the model did not predict such feature for GCs when only a set of EC object-vector cell inputs was added in the cue position but did so when MC activity was altered throughout the whole belt as in the experiment. Hence, an important function of MCs might be to spread the alteration of GC representations in locations distant from the added cue. Because MC-to-GC feed-forward inhibition^77–79^ was not incorporated in the model, MC-to-GC excitation might alone be sufficient to support this function. However, MC-to-GC feed-forward inhibition and other mechanisms such as presynaptic inhibition of EC inputs^27,28,52^, likely also contribute. For instance, the remapping of MCs is expected to generate a remapping of inhibitory inputs to GCs, which should alter GC population activity considering the effects of modulating model inhibition reported in Fig. 7d. In terms of function, the spread of GC remapping to distant locations might support enhanced exploratory behaviors in areas surrounding displaced objects^8,10,80^ and be critical for binding information of novel cues and contingencies that are part of the same event but not spatially contiguous. Future experiments using cell-type specific inactivation might directly test MC contribution to the spatial extent of GC remapping and exploratory behaviors.

## Methods

### Animals

All experiments conformed to the Guide for the Care and Use of Laboratory Animals (NRC 2011). The experimental protocols were approved by the Institutional Animal Care and Use Committee of the Korea Institute of Science and Technology.

Data were collected from 8 mice, 5 DRD2-Cre (B6.FVB(Cg)-Tg(Drd2-cre)ER44Gsat, GENSAT) and 3 POMC-Cre (B6.FVB(Pomc-cre)1LowI/J, The Jackson Laboratory) mice aged between 11 and 12 weeks at the time of recordings. Male mice were used for all experiments to avoid gender-related variability. Mice were housed in groups of 2 or 3 per cage in a vivarium with a 12-h light/dark cycle. Training and recording sessions were conducted during the light cycles.

### Virus injection and preparation for head fixation

Part of the surgery procedure has been described previously^81^. Briefly, under isoflurane anesthesia (supplemented by injections of buprenorphine 0.1 mg/kg, s.c), a viral vector expressing Chronos and GFP (AAV-hSyn-FLEX-Chronos-GFP, UNC vector core) was injected bilaterally into the dorsal dentate gyrus (−2.1 mm anteroposterior; −1.5 mm mediolateral; −1.6 and −1.7 mm dorsoventral; 50 nL per location) using a glass micropipette and custom-made injector (using the Narishige mo-10 manipulator). Two screws were inserted into the skull overlying the cerebellum to serve as ground and reference electrodes. A head plate designed to be conveniently fastened to a metal holder was fixed to the skull using dental cement^31^.

### Behavioral training and apparatus

After a recovery period of 7 days, mice were put under a water restriction scheme (1 ml per day) and trained (1 h session per day for 3 weeks) to run on a treadmill with their head restrained. The treadmill consisted of a 211-cm velvet belt stretched between two 3D printed wheels and was not motorized such that mice moved the belt themselves. Pairs of visual-tactile cues were fixed on the belt. Each cue consisted of a double array (15 to 20-cm-long) of small erect objects lined along the edges of the belt and provided visual-tactile stimulation to both sides of the mice without interfering with their locomotion. Three types of objects were used: ~2-cm-high glue spines, ~2-cm-high shrink tubes, and ~1-cm-high pieces of folded Velcro.

To induce consistent running trajectories, a water reward was delivered through a lick port on each trial (belt revolution) at the same belt position. Mice typically ran 100 to 150 trials over 45-minute sessions after 2 weeks of training.

### Silicon probe with integrated fibers

A 64-channel silicon probe (Neuronexus, Buzsaki64sp) was mounted on a custom made microdrive^31^. Optical fibers were chemically etched on one end (down to 15-µm diameter) and fixed on every other shank of the silicon probe (3 out of 6 shanks) with the fiber tip positions 100 µm above the recording sites^30^. Optical fiber connectors (Precision Fiber Products Inc, single-mode LC ferrule) were fixed on the unetched end of the fibers and cemented to a custom-made support^31^.

### Chronic implantation of the electrode

Detailed procedures for chronic implants of silicon probes were described previously^81^. Briefly, a craniotomy was performed under isoflurane anesthesia. The silicon probe was inserted in the dentate gyrus under microscope supervision and electrophysiological activity monitoring. The electrode was slowly lowered to the granule cell layer of the dentate gyrus, which was detected by the emergence of unit activity following an ~500-μm silent zone below the CA1 pyramidal layer. Then the electrode was retracted 200 μm. The microdrive was cemented to both the skull and head plate. A mixture of bone wax and mineral oil was used to cover the skull opening. The next day, the silicon probe was slowly lowered to the granule cell layer using the microdrive. A plastic cap was used to protect the microdrive/silicon probe assembly.

### Recording sessions

On the recording day, two consecutive sessions were implemented while mice were on the treadmill. In the first session, we delivered multiple light stimuli (single ~50-µW amplitude sine waves of a 100-ms duration every 3 s) during periods when the animal was immobile. In the second session, a landmark was added to the belt after 10 to 15 trials. The object was a 15-cm-long double array of glue spines identical to the one already fixed on the belt (Fig. 3a).

### Anatomy, opsin expression and immunohistochemistry

The mice were anesthetized with isoflurane and perfused transcardially with 4% paraformaldehyde in PBS. The brain was removed and kept overnight in 4% paraformaldehyde, after which it was cut into 100-µm-thick coronal sections using a vibratome (Leica, VT1200S). Brain sections were processed for fluorescent immunohistochemistry^82^. The sections were permeabilized with 1% Triton X-100 in 1X Tris-Buffered Saline (1X TBS: 50 mM Tris, 150 mM NaCl, pH 7.4 adjusted with 1 M HCl) and incubated in blocking solution (5% normal donkey serum (Jackson, 017-000-12) and 0.4% Triton X-100 in 1X TBS). Rabbit anti-calretinin (1:1000; Swant, 7697) and rabbit anti-GluR2/3 (1:100; Millipore, AB1506) were used as primary antibodies (4 °C overnight incubation) for DRD2-Cre mice, while rabbit anti-calbindin-D28k (1:1000; Swant, CB38) was used for POMC-Cre mice. Donkey anti-rabbit Alexa Fluor 594 (1:500–1000; Jackson ImmunoResearch, 711-585-15) was used as a secondary antibody (3-h incubation at room temperature). Slices were washed in TBS and mounted using Vectashield mounting medium with DAPI (Sigma-Aldrich). Images of DAPI, GFP and Alexa 594 fluorescence were acquired separately with a confocal microscope (Nikon A1).

### Behavior control and photostimulation

The forward and backward movement increments of the treadmill were monitored using two pairs of LED and photosensors that read patterns on a disc coupled to the treadmill wheel, while the zero position was implemented by an LED and photosensor couple detecting a small hole on the belt. From these signals, the mouse position was implemented in real time by an Arduino board (Arduino Uno, arduino.cc), which also controlled the valves for the reward delivery. Position, time and reward information from the Arduino board was sent via USB serial communication to a computer and recorded with custom-made LabView (National Instruments) programs.

To deliver the photostimuli, a blue diode laser (Vortran Laser Technology, StradusTM 473) was divided and collimated into 4 optical fibers (Thorlabs HPSC10-CUSTOM) using fiber ports (Thorlabs, PAFA-X-4-A). The optical fibers were connected to the electrodes’ fibers via LC connectors (single mode LC ferrule, Precision Fiber Products Inc). The waveform of the light stimulus was controlled using LabVIEW (National Instruments) and a USB Interface Board (Intan Technologies, RHD2000) communicating with the analog port of the laser.

### Data acquisition and spike sorting

Wideband neurophysiological signals were acquired continuously at 30 kHz on a 250-channel recording system (Intan Technologies, RHD2132 amplifier board with RHD2000 USB Interface Board and custom-made LabView interface). The wideband signals were digitally high-pass filtered (0.8–5 kHz) for spike detection, whereas they were low-pass filtered (0–500 Hz) and downsampled to 1000 Hz for local field potentials (LFPs). Spikes from each shank of the silicon probe were clustered separately with automatic algorithms^83^ followed by manual adjustments in custom-made MATLAB routines implementing spike autocorrelation, cross-correlation and cluster isolation statistics. Only clusters with well-defined cluster boundaries and clear refractory periods were included in the analyses^84^.

### Gamma phase of spikes

The LFP was bandpass filtered between 30–80 Hz. A vector of instantaneous phase was derived using the Hilbert transform. The gamma phase of each spike was interpolated from the vector of the instantaneous phase.

### Implementation of a single-neuron firing rate map

The length of the belt was divided into 100 pixels. For each cell, the number of spikes occurring in each pixel (spike count vector) and the time the animal spent in each pixel (time spent vector) were calculated. Both spike count and time spent vectors were smoothed with a Gaussian function and spike count was divided by time spent to obtain a firing rate map.

### Estimation of cell position on electrode shanks

To estimate the position of a cell with respect to the recording sites of a shank, we assumed that the amplitude of spike signals attenuated as 1/d^2^ (see notes below), where d is the distance of the site to the cell soma, such that the amplitude measured at a given site is as follows:

$${{\rm{a}}}_{{\rm{i}}}={\rm{A}}/{{{\rm{d}}}_{{\rm{i}}}}^{2}$$

where A is the spike amplitude exactly at the cell position. For the several recording sites on one shank, this means the following:

$${\rm{A}}={{\rm{a}}}_{1}\ast {{{\rm{d}}}_{1}}^{2}={{\rm{a}}}_{2}\ast {{{\rm{d}}}_{2}}^{2}={{\rm{a}}}_{3}\ast {{{\rm{d}}}_{3}}^{2}={{\rm{a}}}_{4}\ast {{{\rm{d}}}_{4}}^{2}={{\rm{a}}}_{5}\ast {{{\rm{d}}}_{5}}^{2}\cdots $$

Therefore, to estimate the position of a cell, we simply found the position where this condition was optimally fulfilled. To do this, we divided the volume around each shank in 1-µm3 pixels and calculated the Euclidean distances of recording sites for each pixel.

Then, we defined the value S as follows:

$${\rm{S}}={\sum }_{{\rm{ij}}}|{{\rm{a}}}_{{\rm{i}}}\ast {{{\rm{d}}}_{{\rm{i}}}}^{2}-{{\rm{a}}}_{{\rm{j}}}\ast {{{\rm{d}}}_{{\rm{j}}}}^{2}|$$

where i and j varies to generate all combinations of possible sites. The pixel with the smallest value of S was defined as the cell position.

Note: The electric potential of dipoles attenuate to 1/d^2^ and that of monopoles attenuates by 1/d. We tested the method using both forms and found the resultant cell positions to be very similar.

### Spatial information

Spatial information^85^ was calculated using the following equation:

$${\rm{Information}}\,{\rm{per}}\,{\rm{spike}}={\sum }_{{\rm{i}}}{{\rm{P}}}_{{\rm{i}}}\cdot \frac{{{\rm{R}}}_{{\rm{i}}}}{{\rm{R}}}\cdot {\log }_{2}\cdot \frac{{{\rm{R}}}_{{\rm{i}}}}{{\rm{R}}}$$

where i is the pixel number, P_i_ is the probability of occupancy of pixel i, R_i_ is the mean firing rate in pixel i, and R is the overall mean firing rate.

### Model

Three hundred and seventy-five EC cells (300 grid cells and 75 object-vector cells) were connected to 3000 GCs with synaptic weights initially set randomly and selected from a gamma distribution (shape parameter = 0.008). EC cells had firing fields generated with Gaussian functions (half-width of 15 cm) and included 3 groups of grid cells of different periodicities (70/√2 cm, 70 cm, 70*√2 cm)^86^ and 3 groups of object-vector cells encoding a range of positions within 10 cm from the tube, Velcro and spine cues (Fig. 6a). In addition, 30 MCs provided inputs to GCs, with synaptic weights set randomly and selected from a uniform distribution. MCs had several firing fields generated with Gaussian functions (half-width of 30 cm) of randomly varying amplitude and spread at random position on the belt (Fig. 6a). The overall contribution of MC-to-GC synapses was set to be weaker than that of EC-to-GC synapses by setting a constant γ = 0.5 (Fig. 6b (1)). The relative number of cells roughly matched the ~1:5 to 1:10 EC:GC ratio and the 1:100 MC:GC ratio^14,15^.

The excitation received by a GC in a given belt position was equal to the weighted sum of EC cell activity plus the weighted sum of MC activity (Fig. 6b (1)). The feedback inhibition was set proportional (using a constant α = 0.5) to the summed activity of GCs in a given belt position (Fig. 6b (2)). If the excitation exceeded the inhibition, the activity of the GC was equal to the excitation minus the inhibition; otherwise, the activity was set to zero (Fig. 6b (2)). To induce experience-dependent learning, EC-to-GC synaptic weights were incremented proportionally to the level of co-firing of EC-GC pairs (using a synaptic learning rate constant k), similar to Hebbian forms of long-term synaptic potentiation^54^ (Fig. 6b (3)). Then, the synaptic weights were scaled using a function that pulled the sum of synaptic weights for individual GCs toward normalization at a rate adjusted by a constant ß (Fig. 6b (4)).

The simulation was divided into two stages. In the first stage, 10 iterations were implemented to mimic the 3-week period of familiarization to the belt, which produced single place field representations (Fig. 6c) as previously reported^25,43^. For this stage, the synaptic learning rate was set to k = 20, and synaptic rescaling was set to produce complete synaptic weight normalization (ß = ∞). In the second stage, a new place field was added to the firing pattern of object-vector cells that encoded the spine cue in the position of the added spine cue (Fig. 6c). MC activity was reorganized by changing the amplitude of MC firing fields, with the direction of change randomly chosen and the magnitude of change decreasing exponentially with distance from the added cue to generate a similar profile of MC population vector correlation as observed in the experimental data (Fig. 6a). Five iterations were implemented to reproduce the trial-by-trial changes observed within the recording sessions. For this stage, the synaptic learning rate was reduced to k = 1 to generate small trial-by-trial synaptic learning increments, and synaptic scaling rate was reduced to ß = 3 (a feature we found critical to produce a gradual development of population vector and firing rate). The simulation was run 10 times with different sets of random values to obtain mean and standard error values for population vector correlations.

### Statistical analysis

All statistical analyses were performed in MATLAB (MathWorks). The number of animals and the number of recorded cells were similar to those generally employed. For each distribution, a Kolmogorov-Smirnov test was used to test the null hypothesis that the sample distribution was derived from a standard normal distribution. If normality was uncertain, we used nonparametric tests as stated in the main text or figures. Otherwise, Student’s t-tests were used to test the sample mean. Correlations were computed using the Pearson correlation coefficient. The results were considered significant if the p-value was < 0.05.

## Data Availability

The data that were collected for this study are available upon reasonable request.
