# Author Correction: Dentate granule and mossy cells exhibit distinct spatiotemporal responses to local change in a one-dimensional landscape of visual-tactile cues

**DOI:** 10.1038/s41598-019-55788-2

**Published:** 2019-12-24

**Authors:** Dajung Jung, Soyoun Kim, Anvar Sariev, Farnaz Sharif, Daesoo Kim, Sebastien Royer

**Affiliations:** 1 Department of biological sciences, Korea Advanced Institute of Science and Technology, Daejeon, 34141 Republic of Korea; 2 Center for Functional Connectomics, Korea Institute of Science and Technology, Seoul, 02792 Republic of Korea; 3 Division of Bio-Medical Science and Technology, KIST School, Korea University of Science and Technology, Seoul, 02792 Republic of Korea

Correction to: *Scientific Reports* 10.1038/s41598-019-45983-6, published online 02 July 2019

In the original version of this Article, Affiliations 1, 2 and 3 were not listed in the correct order. The correct affiliations are listed below:

Affiliation 1:

Department of biological sciences, Korea Advanced Institute of Science and Technology, Daejeon, 34141, Republic of Korea.

Affiliation 2:

Center for Functional Connectomics, Korea Institute of Science and Technology, Seoul, 02792, Republic of Korea.

Affiliation 3:

Division of Bio-Medical Science and Technology, KIST School, Korea University of Science and Technology, Seoul, 02792, Republic of Korea.

This error has now been corrected in the PDF and HTML versions of the Article.

